# Temporal regulation of kin recognition maintains recognition-cue diversity and suppresses cheating

**DOI:** 10.1038/ncomms8144

**Published:** 2015-05-28

**Authors:** Hsing-I Ho, Gad Shaulsky

**Affiliations:** 1 Department of Molecular and Human Genetics, Baylor College of Medicine, One Baylor Plaza, Houston Texas 77030, USA

## Abstract

Kin recognition, the ability to distinguish kin from non-kin, can facilitate cooperation between relatives. Evolutionary theory predicts that polymorphism in recognition cues, which is essential for effective recognition, would be unstable. Individuals carrying rare recognition cues would benefit less from social interactions than individuals with common cues, leading to loss of the genetic-cue diversity. We test this evolutionary hypothesis in *Dictyostelium discoideum,* which forms multicellular fruiting bodies by aggregation and utilizes two polymorphic membrane proteins to facilitate preferential cooperation. Surprisingly, we find that rare recognition variants are tolerated and maintain their frequencies among incompatible majority during development. Although the rare variants are initially excluded from the aggregates, they subsequently rejoin the aggregate and produce spores. Social cheating is also refrained in late development, thus limiting the cost of chimerism. Our results suggest a potential mechanism to sustain the evolutionary stability of kin-recognition genes and to suppress cheating.

## Abstract (web summary)

It is unclear how variation in cues that enable recognition of kin and facilitate cooperation is maintained. Here, the authors show that rare variants of *Dictyostelium discoideum* are excluded from aggregates when the potential for social cheating is high, but subsequently rejoin the aggregate and produce spores.

## Graphical abstract

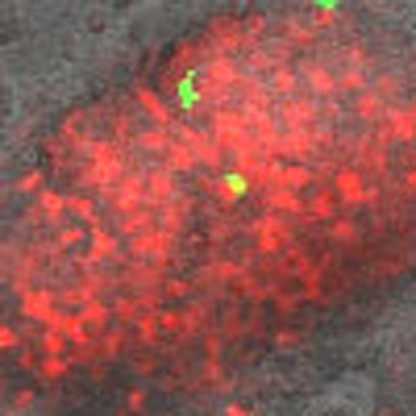

Kin recognition is observed in various organisms^1^,^2^ and the ability to distinguish kin from non-kin can facilitate altruistic behaviours towards relatives and thereby increase inclusive fitness^3^. In genetically based recognition systems, individuals identify kin by matching heritable recognition cues and, therefore, polymorphism in the recognition cues is essential for precise discrimination^1^,^4^,^5^. Paradoxically, kin recognition is predicted to eliminate the very genetic diversity in the recognition-cue loci that is required for its function^3^,^6^,^7^. In social systems, individuals carrying common cues would receive altruistic benefits from matching partners more often than individuals with rare or newly evolved cues^8^,^9^. In addition, individuals with rare cues may incur cost upon aggressive rejection^6^,^10^,^11^,^12^. Consequently, individuals with common cues would become more common in the population due to higher fitness, leading to erosion of polymorphism in the recognition-cue genes and a breakdown of the recognition system^5^,^6^,^7^,^11^.

*Dictyostelium discoideum* are social soil amoebae that aggregate and develop as multicellular organisms upon starvation. During cooperative development, 80% of the cells differentiate into viable spores, whereas the remaining 20% die as stalk cells, altruistically facilitating spore dispersal^13^,^14^. Genetically distinct cells can form chimeric aggregates, leading to potential social conflicts^15^. For instance, cheaters in *D. discoideum* exploit others by producing more spores than their fair share^16^, which is defined as the ratio between the strains at the beginning of development. Cheaters are prevalent in nature^15^,^17^ and could collapse the social system without proper control^18^,^19^. Kin recognition in *D. discoideum* limits cheating through strain segregation^20^. The degree of strain segregation in *D. discoideum* is positively correlated with the overall genetic distance and mediated by two transmembrane proteins, TgrB1 and TgrC1 (refs 21, 22). The *tgrB1* and *tgrC1* genes are highly polymorphic in natural populations, possibly under positive or balancing selection^23^. The sequence dissimilarity of these genes is highly correlated with strain segregation in experiments done with unaltered wild isolates^23^. In the laboratory, cells that are genetically engineered to be only different in their *tgrB1-C1* genes segregate from one another when mixed at equal proportions^21^. These and other results indicate that a compatible *tgrB1-C1* pair is both necessary and sufficient for kin recognition in *D. discoideum*^21^,^22^,^23^.

The maintenance of polymorphism in *tgrB1* and *tgrC1* is baffling because the cost of carrying an uncommon allele is predicted to be high^6^,^7^. Upon starvation, cells with rare *tgrB1-C1* alleles co-aggregate with the majority cell type, in response to the signal molecule cyclic adenosine monophosphate (Fig. 1a,b). They later migrate with reduced speed and directionality and segregate to the periphery of the aggregate (Fig. 1c). In addition, the rare incompatible cells fail to express prespore genes, such as *cotB* (A. Kuspa, personal communication), suggesting that they would be precluded from participation in the fruiting body (Fig.1d). On the basis of evolutionary theory, we hypothesize that rare recognition variants would incur a high cost when cooperating with incompatible cells owing to exclusion from the fruiting bodies. As a result, cells with rare *tgrB1-C1* alleles would not form spores following starvation (Fig. 1e). Interestingly, we find that cells with rare *tgrB1-C1* alleles propagate among other incompatible majority cells at no cost. They generate spores through temporally suppressed kin recognition at a later developmental stage.

## Results

### Cells with rare cues make spores among incompatible cells

To test the hypothesis that rare recognition variants would incur a high cost when cooperating with incompatible cells, we used gene replacement strains, which carry divergent *tgrB1-C1* alleles and segregate well from one another^21^,^22^, to maximize the potential cost of discrimination and to test the system under extreme conditions. The divergent alleles (for example, *tgrB1*^*QS31*^*tgrC1*^*QS31*^) were obtained from wild isolates that segregate from one another. We did not directly use these wild isolates in our experiments because they contain many other uncharacterized genetic differences (∼40,000 SNPs; E. Ostrowski, personal communication). Instead, the gene replacement strains were generated in the AX4 wild-type background, and they only differ in the *tgrB1-C1* locus, thus avoiding the potentially confounding effects of other variable genetic determinants.

**Figure 2:**
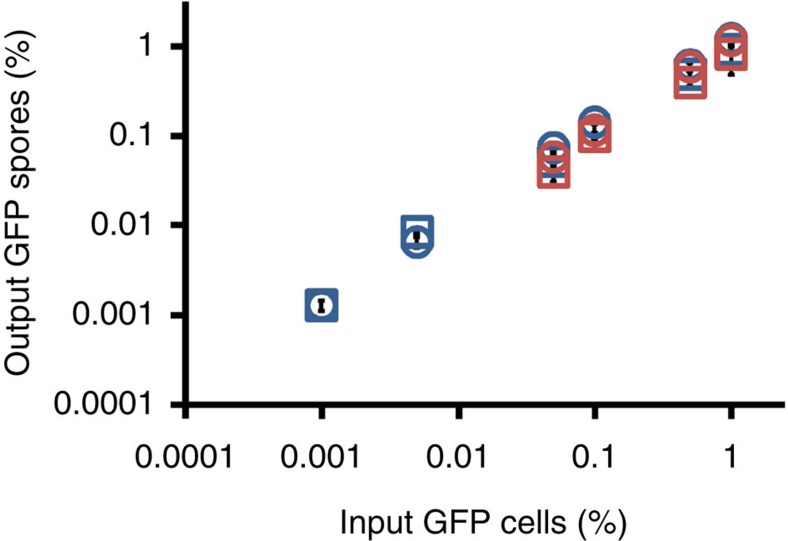
Cells with rare recognition cues produce equal amounts of spores in mixes with either compatible or incompatible strains. We mixed GFP-labelled cells with compatible (control) or incompatible (experiment) unlabelled cells at the indicated frequencies (*x* axis), allowed them to develop, collected the spores and measured the frequency of fluorescent spore at the end of development (*y* axis). Blue squares, rare *tgrB1*^*AX4*^*tgrC1*^*AX4*^–GFP mixed with compatible *tgrB1*^*AX4*^*tgrC1*^*AX4*^ as a control. Blue circles, rare *tgrB1*^*AX4*^*tgrC1*^*AX4*^–GFP mixed with incompatible *tgrB1*^*QS31*^*tgrC1*^*QS31*^. Red squares, rare *tgrB1*^*QS31*^*tgrC1*^*QS31*^–GFP mixed with compatible *tgrB1*^*QS31*^*tgrC1*^*QS31*^ as a control. Red circles, rare *tgrB1*^*QS31*^*tgrC1*^*QS31*^–GFP mixed with incompatible *tgrB1*^*QS38*^*tgrC1*^*QS38*^. The data are means±s.e.m. and both axes are displayed in log_10_ scale. *n*=3–5 per group, two-tailed Student’s *t*-test between controls and experiments at each mixing frequency.

We mixed *tgrB1*^*AX4*^*tgrC1*^*AX4*^–GFP (green fluorescent protein) cells at low frequency with incompatible *tgrB1*^*QS31*^*tgrC1*^*QS31*^, or with compatible *tgrB1*^*AX4*^*tgrC1*^*AX4*^ cells and allowed them to develop. We measured the cost to the *tgrB1*^*AX4*^*tgrC1*^*AX4*^–GFP cells by comparing GFP-positive spore production between the two mixtures. Our hypothesis would be supported if the *tgrB1*^*AX4*^*tgrC1*^*AX4*^–GFP produced fewer or no spores when co-developed with a majority of incompatible *tgrB1*^*QS31*^*tgrC1*^*QS31*^ cells. Surprisingly, we found that at mixing frequencies between 0.05% and 1%, the rare *tgrB1*^*AX4*^*tgrC1*^*AX4*^–GFP cells produced equal amounts of spores, whether they were mixed with compatible or with incompatible cells (Fig. 2, blue symbols). We observed consistent results in reciprocal mixes between a minority of *tgrB1*^*QS31*^*tgrC1*^*QS31*^–GFP and a majority of incompatible *tgrB1*^*AX4*^*tgrC1*^*AX4*^ (Supplementary Fig. 1). In mixes between *tgrB1*^*QS31*^*tgrC1*^*QS31*^–GFP and another incompatible strain, *tgrB1*^*QS38*^*tgrC1*^*QS38*^, we found that *tgrB1*^*QS31*^*tgrC1*^*QS31*^–GFP produced the same amount of spores in both compatible and incompatible mixtures (Fig. 2, red symbols). The reproducibility of the results with different divergent alleles suggests that these findings were not peculiar to one set of alleles.

The input frequencies of fluorescently labelled cells were kept low so they would mostly interact with non-labelled cells during development. We even lowered the frequency of the incompatible cells further, to one GFP-labelled cell per aggregate (a typical aggregate contains 100,000 cells), to further reduce the potential contact between the rare fluorescent cells, and the rare variants still sporulated equally well between mixes with compatible or incompatible cells (Fig. 2b, blue symbols, 0.001%). These results refute our hypothesis and indicate that individuals with rare recognition cues suffer no detectable cost when co-developed with incompatible strains.

### Rare incompatible cells rejoin the group after segregation

**Figure 3:**
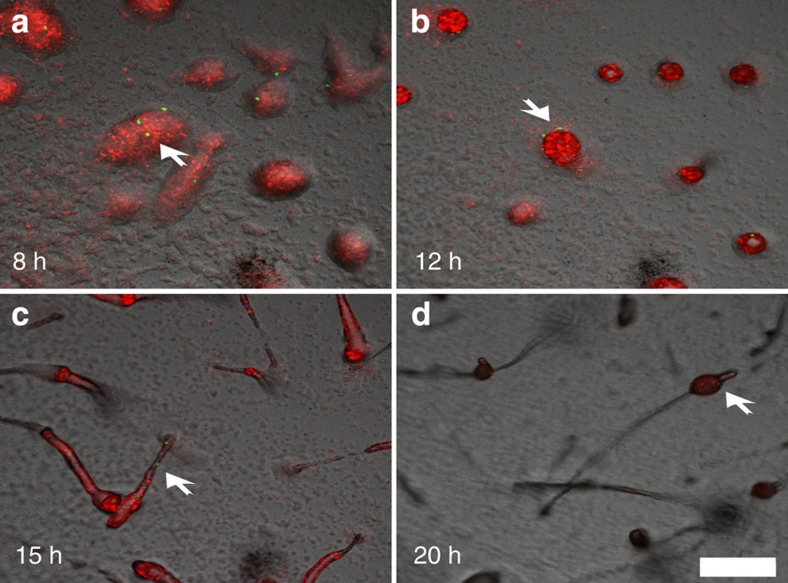
Rare incompatible cells segregate from the majority but eventually rejoin the population and produce spores. We mixed *tgrB1*^*AX4*^*tgrC1*^*AX4*^–GFP cells with incompatible *tgrB1*^*QS31*^*tgrC1*^*QS31*^–RFP at 1:1,000 and allowed them to develop. Multicellular structures were photographed by fluorescent confocal microscopy at a fixed position over the indicated times. (**a**) Loose aggregates. (**b**) Tight aggregates. (**c**) Slugs. (**d**) Fruiting bodies. The white arrows indicate the position of the rare GFP cells. Scale bar, 200 μm.

To investigate how cells with rare allotypes produce spores following segregation from incompatible cells, we mixed 0.1% of *tgrB1*^*AX4*^*tgrC1*^*AX4*^–GFP cells with incompatible *tgrB1*^*QS31*^*tgrC1*^*QS31*^–RFP (red fluorescent protein) cells and traced them throughout development (Supplementary Movie 1). The rare *tgrB1*^*AX4*^*tgrC1*^*AX4*^–GFP cells initially aggregated into loose mounds together with the majority cells (Fig. 3a). The GFP-positive cells subsequently segregated to the periphery of the mound (Fig. 3b), confirming the observation that rare recognition variants do not cooperate with the rest of the cells after initial co-aggregation (A. Kuspa, personal communication). Later in development, the GFP-positive cells were found in slugs (Fig. 3c) and in spore-bearing sori (Fig.). This unexpected observation excludes the possibility that rare incompatible cells produce spores by forming small clonal fruiting bodies after segregation. Instead, it suggests that the initially excluded cells can rejoin the population and participate in spore formation later, regardless of the incompatibility in *tgrB1-C1* genes. We therefore hypothesized that *tgrB1-C1*-mediated kin recognition is diminished in late developmental stages.

### Kin recognition is suppressed in late development

**Figure 4:**
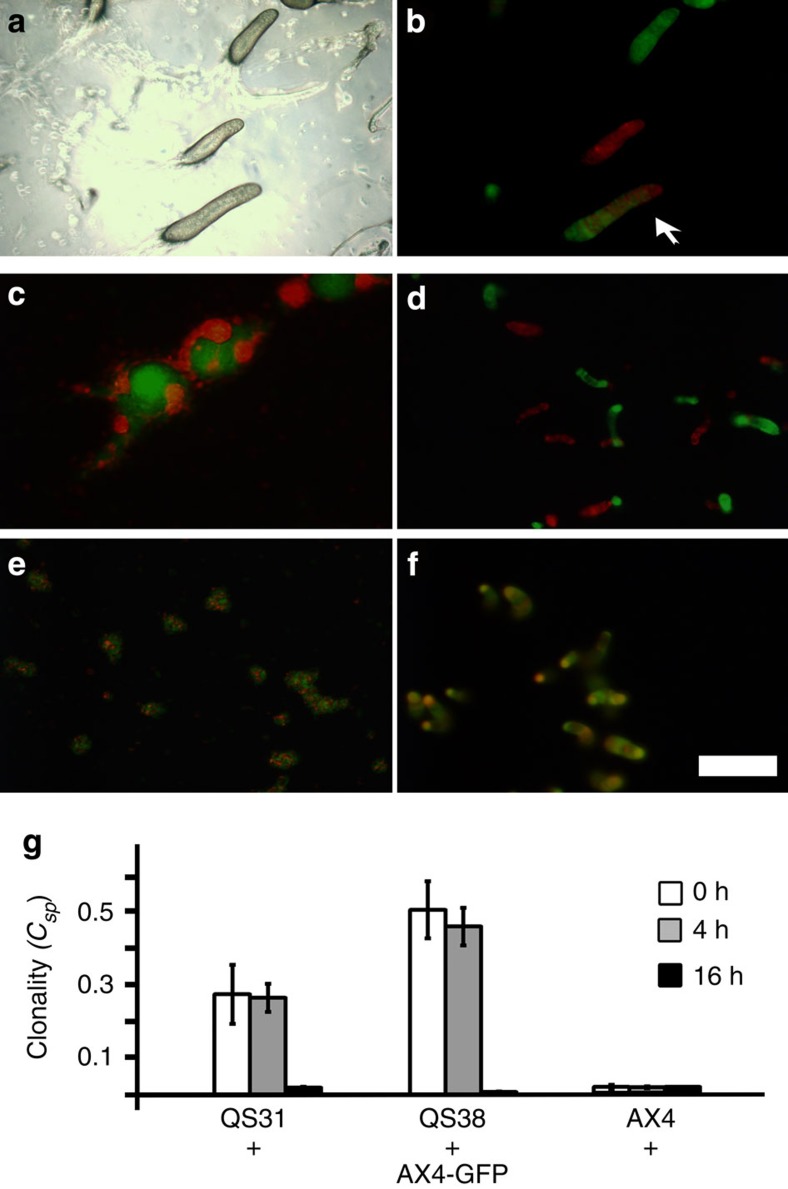
Kin recognition is lost at the slug stage. We developed *tgrB1*^*AX4*^*tgrC1*^*AX4*^–GFP and incompatible *tgrB1*^*QS31*^*tgrC1*^*QS31*^–RFP strains separately on agar plates until the slug stage. (**a**,**b**), Slug merging. We sliced the agar and reassembled different slices to bring slugs from different strains into close proximity. The slugs were then prompted to migrate toward unidirectional light. We photographed a fixed position of the resulting slugs with light (**a**) and fluorescence (**b**) microscopy. The arrow indicates a merged slug (b). (**c**–**f**) Cell mixing. We developed pure populations of the same strains as above, disaggregated them at different developmental times, mixed the two dissociated strains at equal proportion and allowed them to develop again. We photographed the multicellular structures with fluorescence microscopy. (**c**,**d**) The cells were dissociated at 4 h and photographed 7 h (**c**) and 14 h (**d**) after re-association. (**e**,**f**) The cells were dissociated at 16 h and photographed 1 h (**e**) and 4 h (**f**) after re-association. Scale bar, 200 μm. (**g**) Spore production. We developed the strains separately, disaggregated them at the indicated times, mixed the disaggregated strains, developed them and collected spores from individual fruiting bodies. We quantified the GFP-positive spores and calculated the clonality increase of individual fruiting bodies solely owing to segregation (*C*_sp_). The spore genotypes are indicated on the *x* axis where *tgrB1*^*AX4*^*tgrC1*^*AX4*^–GFP (AX4-GFP) was mixed with the incompatible strains *tgrB1*^*QS31*^*tgrC1*^*QS31*^ (QS31) and *tgrB1*^*QS38*^*tgrC1*^*QS38*^ (QS38), or with the compatible strain *tgrB1*^*AX4*^*tgrC1*^*AX4*^ (AX4). The bars (clonality (*C*_sp_)) represents the ability to segregate where 0 indicates no segregation and 1 indicates complete segregation between two strains; the shading indicates the times at which the clonally developed strains were disaggregated and mixed. The data are means±s.e.m., *n*=3 per group, where each replica represents 20–30 single fruiting bodies.

To evaluate the efficacy of kin recognition in late development, we first tested it during slug migration. Two incompatible strains, *tgrB1*^*AX4*^*tgrC1*^*AX4*^–GFP and *tgrB1*^*QS31*^*tgrC1*^*QS31*^–RFP, were developed separately until they formed slugs. We then brought the slugs into close proximity and allowed migration under conditions that promote slug merging^24^. We found slugs containing mixed GFP- and RFP-labelled cells (Fig. 4a,b), indicating that slugs can merge despite the *tgrB1-C1* incompatibility and suggesting that kin discrimination is lost in late development.

To further examine the loss of kin discrimination, we clonally developed the incompatible strains *tgrB1*^*AX4*^*tgrC1*^*AX4*^–GFP and *tgrB1*^*QS31*^*tgrC1*^*QS31*^–RFP. We disaggregated the cells at different stages, mixed them at equal proportions and allowed them to redevelop. Strains that were disaggregated after 4 h of development segregated from each other at the streaming stage (Fig.) and eventually formed nearly clonal fingers (Fig. 4d). These results were identical to the ones reported when strains were co-developed without disaggregation^21^, indicating that the kin-recognition system functions at 4 h of development and that our experimental treatment did not disrupt it. When disaggregated at 16 h and then mixed, the strains did not segregate but rather formed mixed multicellular structures shortly after mixing (Fig. 4e) and mixed slugs later on (Fig. 4f), suggesting that the *tgrB1-C1* system was not functional at 16 h of development.

To quantify segregation, clonally developed *tgrB1*^*AX4*^*tgrC1*^*AX4*^–GFP cells, incompatible *tgrB1*^*QS31*^*tgrC1*^*QS31*^ and *tgrB1*^*QS38*^*tgrC1*^*QS38*^ cells and compatible *tgrB1*^*AX4*^*tgrC1*^*AX4*^ cells were disaggregated at different stages. Disaggregated GFP cells were mixed with unlabelled strains in pairwise combinations and redeveloped. We quantified the proportion of GFP-labelled spores in individual sori and calculated the increase in clonality^25^. We found that mixing vegetative cells (0 h) or cells disaggregated at 4 h gave similar results. The incompatible strains *tgrB1*^*QS31*^*tgrC1*^*QS31*^ and *tgrB1*^*QS38*^*tgrC1*^*QS38*^ segregated from *tgrB1*^*AX4*^*tgrC1*^*AX4*^–GFP, whereas the compatible *tgrB1*^*AX4*^*tgrC1*^*AX4*^ cells did not (Fig. 4g). At 16 h, however, all the strains mixed equally well regardless of their allotypes. These results further support the hypothesis that kin recognition is lost at the slug stage.

To test the broader applicability of our findings, we used four natural isolates, QS4, QS31, NC34.1 and NC105.1 (refs 21, 23, 26, 27), in the same experimental system. All wild isolates segregated well from each other at 0 h (Supplementary Fig. 2, 0 h). However, they mixed evenly when all the strains were first developed clonally for 16 h and then allowed to mix (Supplementary Fig. 2, 16 h). These results suggest that the loss of kin recognition at the slug stage is also true among wild isolates.

### Cheating is also limited during late development

**Figure 5:**
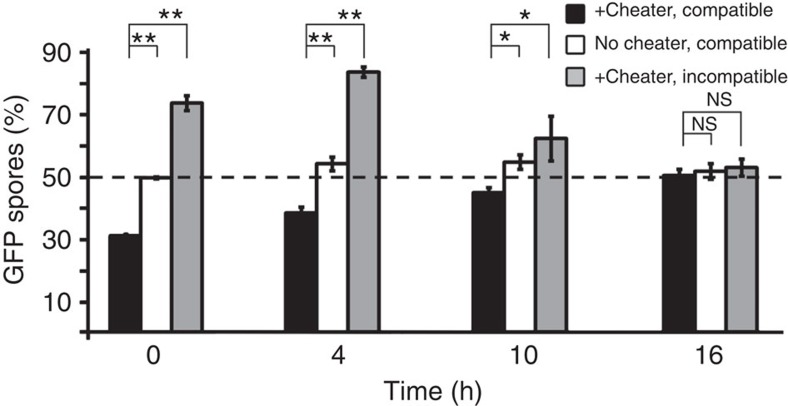
Cheating and kin recognition are diminished during development. We developed cells in pure populations, dissociated them at different times as indicated (*x* axis), mixed them at equal proportions and allowed them to develop again. The test victim was labelled with GFP. We harvested the spores and calculated the proportion (%) of GFP-positive spores (*y* axis). The bars represent the means of three to five independent experiments. Black, *tgrB1*^*AX4*^*tgrC1*^*AX4*^–GFP mixed with the compatible cheater *fbxA*^*−*^; White, *tgrB1*^*AX4*^*tgrC1*^*AX4*^–GFP mixed with the compatible *tgrB1*^*AX4*^*tgrC1*^*AX4*^ strain as a control; Grey, *tgrB1*^*QS31*^*tgrC1*^*QS31*^–GFP mixed with the incompatible cheater *fbxA*^*−*^. The dashed line represents a fair share of spore representation (50%). The data are means±s.e.m., *n*=3–5 per group, *P<0.05, ^**^P<0.001, NS *P*>0.1, two-tailed Student’s *t*-test.

The reduction in kin recognition during late development suggests that incompatible cheaters could rejoin the population and threaten the cooperators, which would seem inconsistent with our previous finding that kin recognition protects against cheaters^20^. We therefore assessed cheating at different developmental stages using the disaggregation–re-association method. We used *fbxA*^*−*^, one of the strongest cheaters in the AX4 genetic background^19^, compatible *tgrB1*^*AX4*^*tgrC1*^*AX4*^–GFP and incompatible *tgrB1*^*QS31*^*tgrC1*^*QS31*^–GFP. We grew and developed these strains in clonal populations, disaggregated them at different times, made pairwise mixes in equal proportions and redeveloped them. We estimated cheating by quantifying the proportion of the GFP-labelled spores (Fig. 5). We found that among cells disaggregated at 0 and 4 h, *fbxA*^*−*^ cheated on the compatible *tgrB1*^*AX4*^*tgrC1*^*AX4*^–GFP, but not on the incompatible *tgrB1*^*QS31*^*tgrC1*^*QS31*^–GFP cells, confirming that kin recognition protects from cheaters during early development. At 16 h, *fbxA*^*−*^ and both the compatible *tgrB1*^*AX4*^*tgrC1*^*AX4*^–GFP and the incompatible *tgrB1*^*QS31*^*tgrC1*^*QS31*^–GFP cells made 50% of the spores, suggesting cheating by *fbxA*^*−*^ was restrained at late stages. At 10 h, segregation between the incompatible strains (Supplementary Fig. 3) and cheating by *fbxA*^*−*^ (Fig. 2) were both reduced compared with 4 h, suggesting an intermediate state between the early and late developmental stages. In the controls (Fig. 5, white bars), *tgrB1*^*AX4*^*tgrC1*^*AX4*^–GFP and *tgrB1*^*AX4*^*tgrC1*^*AX4*^ produced equal amounts of spores at all times, indicating that the experimental procedure did not perturb normal sporulation.

We also observed developmental regulation of cheating in two other strains that utilize different cheating strategies^16^,^17^ (Supplementary Fig. 4), suggesting that cheating of several independent cheaters is suppressed at late development, when kin recognition is also diminished.

## Discussion

We have found that the *tgrB1-C1*-mediated recognition is temporally regulated—it is active during aggregation and suppressed at later developmental stages. One possible explanation for the temporal suppression of kin recognition is the loss of *tgrB1-C1* expression. Both *tgrB1* and *tgrC1* exhibit their highest RNA abundance around the time of aggregation and these levels decline between 12 and 16 h of development^23^, which correlates with the temporal regulation of kin recognition. In addition, the effective timing of kin recognition overlaps with cheating, which could be evolutionarily advantageous because kin recognition protects against cheating in *Dictyostelium*^20^. In all the cheaters we have tested, cheating was suppressed at late development. This observation could possibly result from reduced cheating ability or from a limited time window for cheating, which could be a new aspect for further understanding or characterization of cheating mechanisms.

Obligatory cheaters like *fbxA*^*−*^ cannot sporulate in clonal populations, so their propagation is predicted to be self-limiting^19^,^28^. Our results suggest that the cooperative benefit (sporulation) can be uncoupled from cheating and that cheaters can alter their social behaviour at different developmental times, providing a potential strategy to reduce self-limitation.

Chimerism has both costs and benefits^5^,^29^,^30^,^31^. Fusion between conspecific individuals could lead to an advantage in the form of a larger group size, but it could also lead to conflicts between the participants^5^,^32^. In *D. discoideum,* the costs include exposure to cheaters and increased contribution to the stalk^15^. The benefits include prolonged slug migration and improved spore dispersal^14^,^30^. Kin recognition reduces the costs of chimerism, but constitutive expression of kin-recognition cues could be costly. We propose that a kin-recognition system that functions during early development enables the cells to remain largely clonal while prespore/prestalk differentiation takes place^33^. As development continues and the threat of cheating is reduced, kin recognition is diminished and chimerae can form. Therefore, temporal regulation of kin recognition allows *D. discoideum* to minimize the perils while maximizing the benefits of chimerism.

Genetically based recognition systems are predicted to be evolutionary unstable because of the difficulty in maintaining cue diversity^6^,^7^,^34^. Several solutions have been proposed, including limited dispersal^9^, disassortative mating^35^ or additional balancing selection^6^,^36^ such as host–pathogen interactions^37^. We provide another potential solution to preserving genetic diversity in recognition cues through temporal regulation of the kin-recognition system. As demonstrated here, cells with rare recognition alleles are segregated first, but they are capable of rejoining and cooperating with the majority strains to complete development. Due to loss of kin recognition at later developmental stages, they suffer no reproductive cost in spore production and are able to maintain their frequencies within the populations. Conditionally regulated kin recognition has been suggested in other systems^5^,^38^,^39^,^40^, and it could potentially facilitate the spread of rare recognition variants as we have described here.

## Methods

### Cell growth and development

We grew the cells (Supplementary Table 1) in shaking suspension in HL5 medium to mid-logarithmic state. To begin development, we collected the cells and washed them in KK2 buffer (14 mM KH_2_PO_4_, 3.4 mM K_2_HPO_4_, pH=6.4). We then deposited them on buffer-soaked nitrocellulose membranes or on 2% agar plates made in KK2 buffer. Wild isolates were grown on nutrient agar plates in association with *Klebsiella pneumoniae* instead of HL5. All the double gene replacement strains were *ura*^−^, so the growth medium was supplemented with 20 μg ml^−1^ uracil. We added 10 μg ml^−1^ G418 as necessary for selecting fluorescent protein expression, and removed the drug at least 24 h before development.

### Real time photography of *D. discoideum* development

Cells were developed on six-well KK2 agar plates. We photographed the multicellular structures by confocal fluorescence microscopy at a fixed position every 10 min between 7 and 23 h of development. The movie (Supplementary Movie 1) was produced from the resulting pictures. We used pictures taken from different vertical positions to reach optimal resolution.

### Slug merging experiment

Differentially fluorescence-labelled strains were developed on KK2 agar separately until the early slug stage. We sliced the agar into quarters and reassembled the slices such that slugs of different strains were brought to close proximity. Subsequent slug migration was promoted by unidirectional light for a few hours, after which we photographed the slugs by direct light and fluorescence microscopy.

### Strain segregation experiment

Different strains were mixed at the indicated proportions at a density of 1 × 10^7^ cells ml^−1^ in PDF buffer and deposited in 40 μl drops on a 5-cm KK2 agar plate. We incubated the cells in a dark humid chamber. Photographs were taken at the streaming stage (8–12 h) and the slug stage (14–16 h) with fluorescence microscopy.

### Disaggregation and re-association of multicellular structures

Cells were developed in clonal populations on KK2 agar. We collected cells at the indicated times in KK2 buffer with 20 mM EDTA. At times 10 and 16 h, we collected multicellular structures by filtration through a 40-μm cell strainer to exclude any remaining single cells and obtain multicellular structures for subsequent disaggregation. Cells were disaggregated by trituration in KK2 buffer with 20 mM EDTA and then filtered through a 40-μm cell strainer to eliminate the remaining multicellular structures. We washed the cells three times with KK2 buffer to remove EDTA and allowed them to re-associate and continue development.

### Quantification of segregation

Strains were mixed and allowed to develop into fruiting bodies. To quantify segregation, we collected individual sori with 10 μl pipette tips. We resuspended the spores in KK2 buffer with 0.1% NP40 to eliminate any amoebae. We measured the proportion of GFP-positive spores within individual sori by the Attune Acoustic Focusing Cytometer. We calculated the increase in clonality solely due to segregation out of the maximum possible (*C*_sp_) as follows by adapting the procedure described in ref. 25.

We assessed fruiting body clonality by measuring the presence of one or two clones within individual sori. We calculated the average clonality of all the fruiting bodies in a mixing experiment in equation (1):




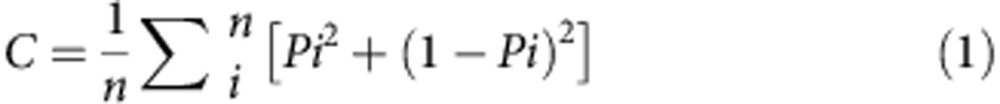




where *C* represents the average clonality, *Pi* is the proportion of the GFP-labelled strain in sorus *i*, (1−*Pi*) is the proportion of the non-labelled strain in the same sorus and *n* is the number of sori sampled.

In each instance, we mixed two strains in equal proportions at the onset of development, so the average clonality would be 0.5 if each strain produced half of the spores in every fruiting body. Increased clonality could result from two factors, segregation and cheating. We estimated these factors in equations (2) and (3):




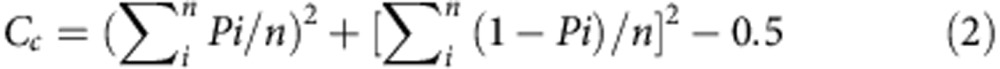




where *C*_*c*_ represents the increase in clonality owing to cheating. In the absence of strain segregation, if *Pi*≠0.5, *C*_*c*_ would be >0, indicating that some of the clonality increase was caused by cheating; and




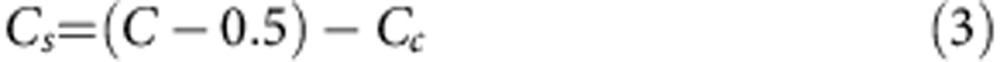




where *C*_*s*_ represents the amount of increased clonality owing to segregation, (*C*−0.5) is the increase in clonality after development and *C*_*c*_ is as defined in equation (2). *C*_*s*_ measures the increase in clonality owing to segregation and removes the effects of cheating. However, the amount of segregation can be confined by cheating. For example, if one strain completely exploits the other and produces all the spores, clonality would be 1, but the increase would be entirely due to cheating, resulting in *C*_*s*_=0. To better estimate segregation, we calculated the clonality increase due to segregation out of the maximum possible after cheating as shown in equation (4):




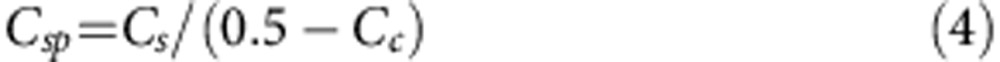




where *C*_*sp*_ represents the ability to segregate while removing the possible effects of cheating on clonality increase. *C*_*sp*_ values range from 0 to 1, where 0 indicates no segregation and 1 indicates complete segregation between two strains.

## Additional information

**How to cite this article**: Hsing-I Ho & Gad Shaulsky. Temporal regulation of kin recognition maintains recognition-cue diversity and suppresses cheating. *Nat. Commun.* 6:7144 doi: 10.1038/ncomms8144 (2015).

## Supplementary Material

Supplementary Figures, Tables and References Supplementary Figures 1-4, Supplementary Table 1 and Supplementary References

Supplementary Movie 1 Rare incompatible cells segregate from the majority but rejoin the slug and form spores. We mixed *tgrB1*^*AX4*^*tgrC1*^*AX4*^–GFP cells with incompatible *tgrB1*^*QS31*^*tgrC1*^*QS31*^–RFP at 1:1000 and allowed them to develop. Multicellular structures were photographed by fluorescent confocal microscopy at a fixed position every 10 minutes between 7 and 23 hours of development. The movie was produced from the resulting pictures and still frames were used to generate Fig. 3.

## Figures and Tables

**Figure 1:**
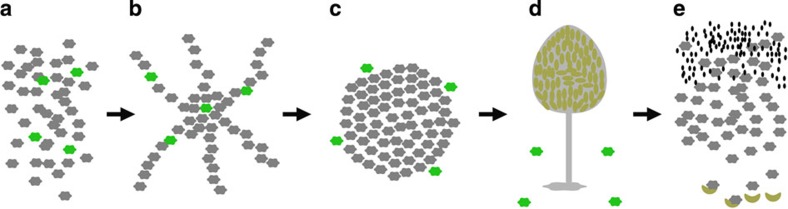
An illustration of the proposed cost to cells that carry rare recognition cures in co-development with incompatible strains. (**a**) Starvation of vegetative cells. The hexagons represent cells; grey—cells with common recognition cues, green—cells with rare, incompatible recognition cues. (**b**) Aggregation—the cells stream toward a central source of cAMP but the recognition cues have no effect yet. (**c**) The onset of multicellularity. Rare incompatible cells are segregated from the majority and excluded to the periphery of the mound. (**d**) Fruiting body—the dark green ellipses represent spores after development. On the basis of our hypothesis, we proposed that the incompatible cells would be excluded from the fruiting body. (**e**) Spore germination—the small black ellipses represent bacteria, which are consumed by the amoebae as they hatch from the spores. Cells with uncommon recognition cues have suffered a reproductive cost following segregation and are eliminated from the population.
