# Supplementary material for: Temporal regulation of kin recognition maintains recognition-cue diversity and suppresses cheating
Source: Nat Commun. 2015 May 28;6:7144. doi: 10.1038/ncomms8144 (PMC4448137; doi:10.1038/ncomms8144)
Supplement: Supplementary Figures, Tables and References — Supplementary Figures 1-4, Supplementary Table 1 and Supplementary References [file ncomms8144-s1.pdf]

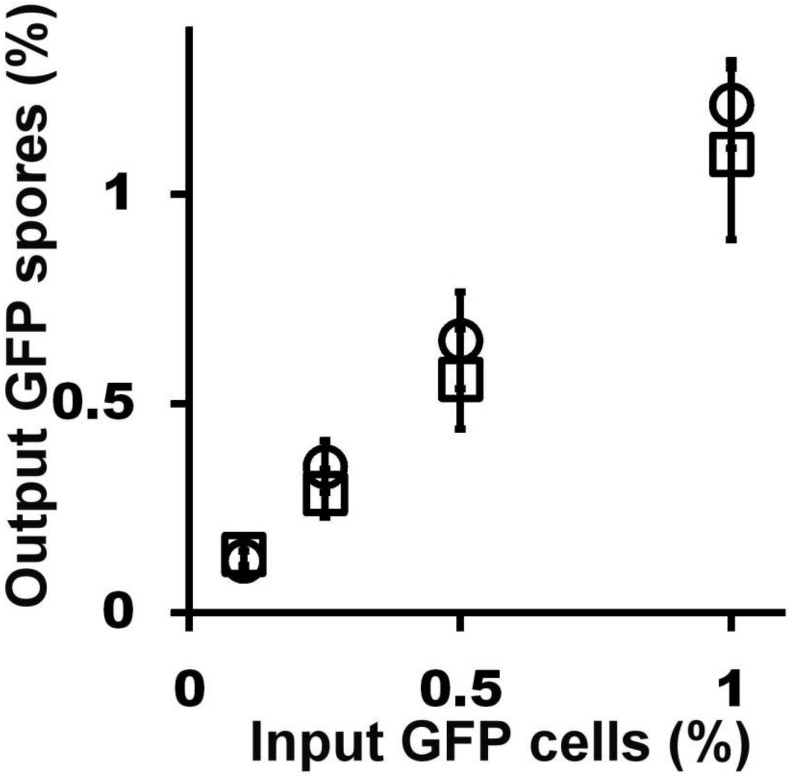

Supplementary Figure 1. ***tgrB1^{QS31}tgrC1^{QS31}* suffered no reproductive costs when co-developed with a majority of incompatible *tgrB1^{AX4}tgrC1^{AX4}*.** We mixed GFP-labeled *tgrB1^{QS31}tgrC1^{QS31}* at the indicated frequencies (x-axis) with compatible *tgrB1^{QS31}tgrC1^{QS31}* (control, squares) or incompatible *tgrB1^{AX4}tgrC1^{AX4}* (experiment, circles) cells and allowed them to develop. We collected the spores and measured the fluorescent spore percentages at the end of development (y-axis). The data are means +/– s.e.m. n= 5 per group, two-tailed student's t-test between controls and experiments at each mixing frequency.

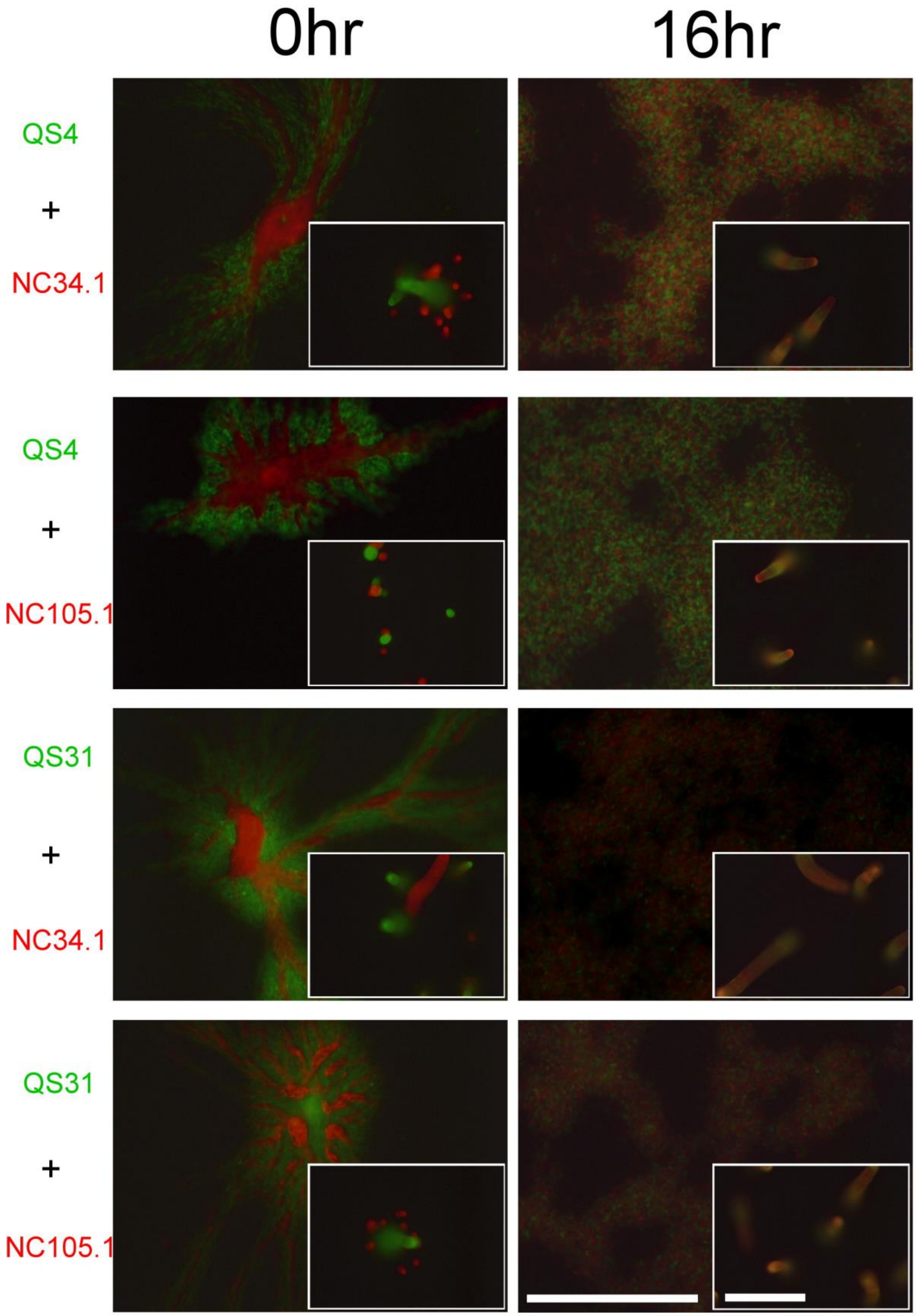

0hr
16hr
QS4
+
NC34.1
QS4
+
NC105.1
QS31
+
NC34.1
QS31
+
NC105.1

Supplementary Figure 2. Kin recognition is diminished at late developmental stages in wild isolates as well. We developed GFP-labeled wild isolates QS4 and QS31and RFP-labeled wild isolates NC34.1 and NC105.1[1,2] in clonal populations, dissociated them at the indicated times after development, mixed the GFP-labeled strains at equal proportions in pairwise combinations with the RFP-labeled strains as indicated and allowed them to develop again. We photographed the multicellular structures at the aggregation stage (main pictures) and the slug stage (inserts) with fluorescence microscopy. The results show that wild isolates with heterogeneous genetic backgrounds segregate from each other during early developmental stages (0hr, first column). Kin recognition was diminished at the later stages, as the wild strain cells are seen to be mixed evenly with each other if the cells were disaggregated at 16 hours after development (second column). Bar = 500µm.

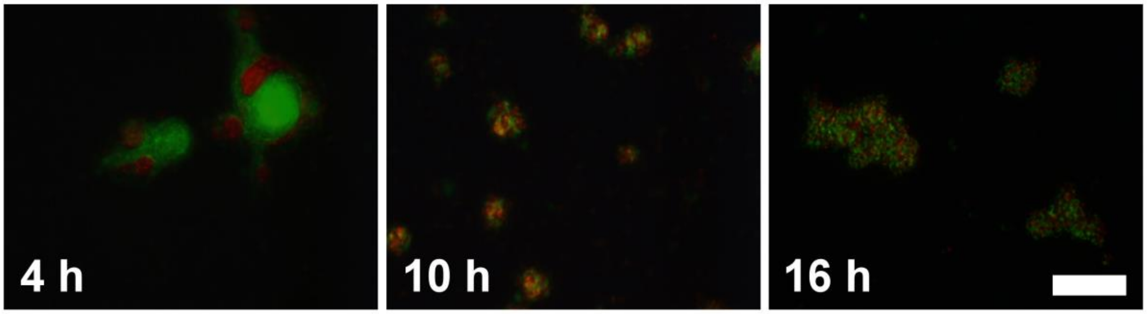

Supplementary Figure 3. Kin recognition is diminished as development progresses. We developed *tgrB1*^{AX4}*tgrC1*^{AX4}–GFP and incompatible *tgrB1*^{QS31}*tgrC1*^{QS31}–RFP separately in clonal populations. We disaggregated the two strains at 4, 10 and 16 hours after development, as indicated, mixed them at equal proportions and allowed them to develop again. We photographed the multicellular structures with fluorescence microscopy 7, 4, and 1 hours after reassociation, respectively. The results show that when disaggregated at 4 hours the strains segregated, at 16 hours they mixed evenly and at 10 hours they exhibited an intermediate behavior. Bar = 200µm.

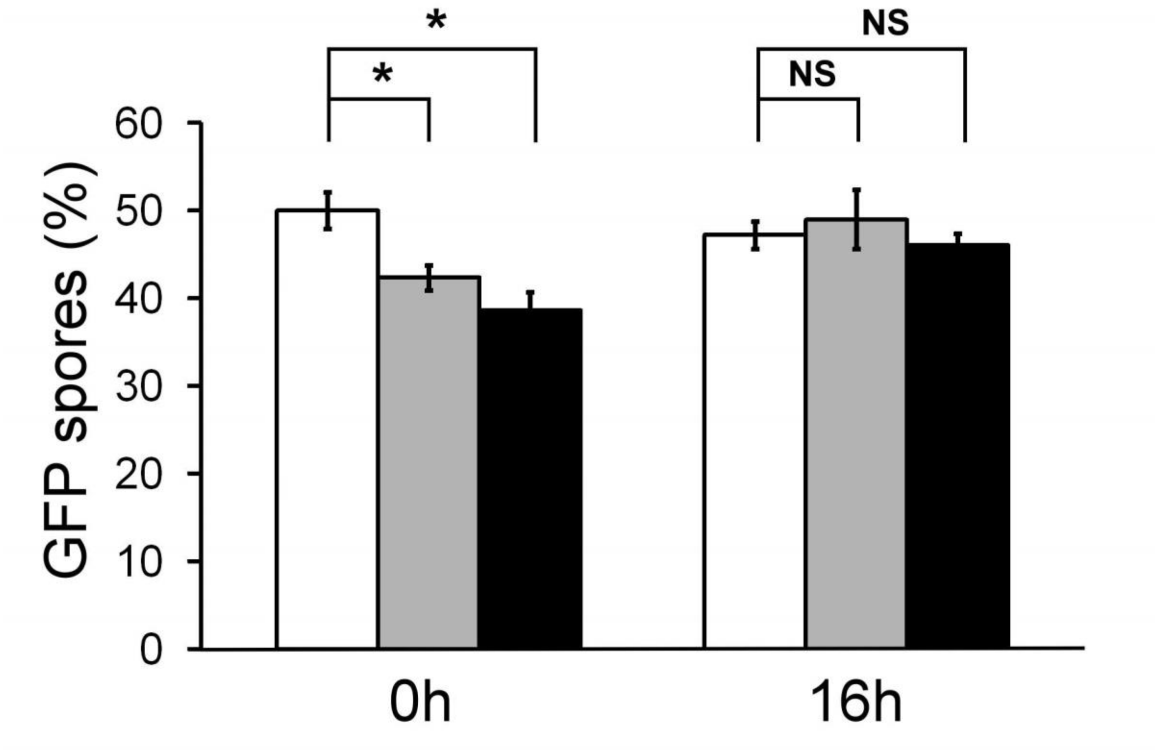

Supplementary Figure 4. Cheating is diminished at late development in multiple cheaters. We developed cells in pure populations, dissociated them at different times as indicated (x-axis), mixed them at equal proportions and allowed them to develop again. The test victim was AX4-GFP. We harvested the spores and calculated the proportion (%) of GFP-positive spores (y-axis). The bars represent the means of 3 independent experiments and bar colors indicate the tested cheater strain: white – control AX4; grey – cheater strains LAS43 and black – cheater strain LAS99. The results show cheating in the 0-hour mixes but no social exploitation by LAS43 and LAS99 at 16hrs of development. Data are means +/– s.e.m., * p<0.05, NS p>0.1, two-tailed student's t-test.

Supplementary Table 1. Strains used in this study

Strain name	Relevant genotype	Parental strain	Used here	Reference number
AK1543	AX4 *tgrB1*^{AX4}/*tgrC1*^{AX4}, *ura*$^{-}$	AK1540	Figure 2, 4, 5, Supplementary Fig. 1	[3]
AK1544	AX4 *tgrB1*^{AX4}/*tgrC1*^{AX4}/ *[act15]*:GFP, *ura*$^{-}$, neoR	AK1543	Figure 2, 3, 4, 5, Supplementary Fig. 3	[3]
AK1549	AX4 *tgrB1*^{QS31}/*tgrC1*^{QS31}, *ura*$^{-}$	AK1540	Figure 2, 4, Supplementary Fig. 1	[3]
AK1550	AX4 *tgrB1*^{QS31}/*tgrC1*^{QS31}/ *[act15]*:GFP, *ura*$^{-}$, neoR	AK1549	Figure 2, 5, Supplementary Fig. 1	[3]
AK1551	AX4 *tgrB1*^{QS31}/*tgrC1*^{QS31}/ *[act15]*:tdTomato, *ura*$^{-}$, neoR	AK1549	Figure 3, 4, Supplementary Fig. 3	[3]
AK1552	AX4 *tgrB1*^{QS38}/*tgrC1*^{QS38}, *ura*$^{-}$	AK1540	Figure 2, 4	[3]
AK1602	AX4 *fbxA*$^{-}$[pBSR3], bsR, *tgrB1*^{AX4}/*tgrC1*^{AX4}, *ura*$^{-}$	AK1543	Figure 5	[4]
AK1503	QS4 *[act15]*:GFP, neoR	QS4	Supplementary Fig. 2	[3]
AK1504	QS31 *[act15]*:GFP, neoR	QS31	Supplementary Fig. 2	[3]
NC34.1–RFP	NC34.1 *[act15]*:RFP, neoR	NC34.1	Supplementary Fig. 2	[1]
NC105.1–RFP	NC105.1 *[act15]*:RFP, neoR	NC105.1	Supplementary Fig. 2	[2]
AX4	Laboratory wild type		Supplementary Fig. 4	[5]
AX4–GFP	AX4 *[act15]*:GFP, neoR	AX4	Supplementary Fig. 4	[5]

LAS43	AX4 *DDB_G0274431*$^{-}$ [pBSR1], bsR	AX4	Supplementary Fig. 4	[5]
LAS99	AX4 *dhkE*$^{-}$[pBSR1], bsR	AX4	Supplementary Fig. 4	[5]

Supplementary References:

1 Francis, D. & Eisenberg, R. Genetic structure of a natural population of Dictyostelium discoideum, a cellular slime mould. *Mol Ecol* **2**, 385-391 (1993).

2 Li, S. I., Buttery, N. J., Thompson, C. R. & Purugganan, M. D. Sociogenomics of self vs. non-self cooperation during development of Dictyostelium discoideum. *BMC genomics* **15**, 616 (2014).

3 Hirose, S., Benabentos, R., Ho, H. I., Kuspa, A. & Shaulsky, G. Self-recognition in social amoebae is mediated by allelic pairs of tiger genes. *Science* **333**, 467-470 (2011).

4 Ho, H. I., Hirose, S., Kuspa, A. & Shaulsky, G. Kin recognition protects cooperators against cheaters. *Curr Biol* **23**, 1590-1595 (2013).

5 Santorelli, L. A. *et al.* Facultative cheater mutants reveal the genetic complexity of cooperation in social amoebae. *Nature* **451**, 1107-1110 (2008).
